# A Lorentz Force EMAT Design with Racetrack Coil and Periodic Permanent Magnets for Selective Enhancement of Ultrasonic Lamb Wave Generation

**DOI:** 10.3390/s23010096

**Published:** 2022-12-22

**Authors:** Xinfeng Guo, Wujun Zhu, Xunlin Qiu, Yanxun Xiang

**Affiliations:** Shanghai Key Laboratory of Intelligent Sensing and Detection Technology, School of Mechanical and Power Engineering, East China University of Science and Technology, Shanghai 200237, China

**Keywords:** electromagnetic acoustic transducer, ultrasonic Lamb waves, selective enhancement, periodic permanent magnets, Lorentz force, antisymmetric mode

## Abstract

This article proposes an electromagnetic acoustic transducer (EMAT) for selectively improving the purity and amplitude of ultrasonic Lamb waves in non-ferromagnetic plates. The developed EMAT consists of a racetrack coil and a group of periodic permanent magnets (PPMs). Two-dimensional finite element simulations and experiments are implemented to analyze the working mechanism and performance of the PPM EMAT. Thanks to the specific design, the eddy currents increase with increasing wire density and the directions of the magnetic fields and Lorentz forces alternate according to the polarities of the magnet units. Wires laid uniformly beneath the magnets, and the gaps between adjacent magnets generate tangential and normal Lorentz forces, resulting in-plane (IP) and out-of-plane (OP) displacements, respectively. The constructive interference occurs when the wavelength of the generated Lamb wave is twice the spacing of the magnets, leading to large amplitudes of the targeted ultrasonic Lamb waves. Therefore, the PPM EMAT is capable of generating pure symmetric or antisymmetric mode Lamb waves at respective frequencies. The results prove that the developed PPM EMAT can generate pure either S0 or A0 mode Lamb waves at respective frequencies. The increase in wire width and wire density further increases the signal amplitudes. Compared with the case of conventional meander-line-coil (MLC) EMAT, the amplitudes of the A0 and S0 mode Lamb waves of our PPM EMAT are increased to 880% and 328%, respectively.

## 1. Introduction

Due to the low attenuation and long propagation distance, ultrasonic Lamb waves are widely used in non-destructive evaluation to ensure the safety and stability of industrial equipment [1,2,3,4,5]. According to the symmetry of the particle displacement relative to the mid-plane of the plate, the modes of ultrasonic Lamb waves are classified into symmetric (Sn) modes and antisymmetric (An) modes [6]. The fundamental Lamb wave modes, S0 and A0 modes, are widely used for plate-like structures because of their simple wave structures and less dispersion at the low frequency-thickness product [7]. The S0 mode outperforms the A0 mode for the detection of through-thickness cracks with the pulse-echo method due to the stronger reflection of the S0 mode [8]. In contrast, the A0 mode is more suitable for the detection of surface cracks with the pulse-catch method than the S0 mode [9].

Ultrasonic Lamb waves are typically generated and received by means of piezoelectric transducers (PZTs) because of their high efficiency. However, PZTs require physical contact with the sample surface, and the generation of ultrasonic waves requires wedges to convert the ultrasonic waves into Lamb-Rayleigh waves [10]. This restricts the application on a curved surface or in high-temperature environments [11,12,13,14]. Electromagnetic acoustic transducers (EMATs) can directly generate ultrasonic waves in conductive materials without direct contact or coupling. Through the different combinations of coils and magnets, different modes of ultrasonic waves can be generated and received in metal specimens [15]. These feathers render EMATs capable of generating and receiving ultrasonic waves in high-temperature environments and on rough or corroded surfaces [16,17,18,19,20].

However, compared with PZTs, EMATs have relatively low energy transfer efficiency, especially when testing non-ferromagnetic materials [21,22,23]. This leads to a low signal-to-noise ratio (SNR) and difficulties in the signal analysis [24,25]. Therefore, lots of research focus on the improvement of the signal-to-noise ratio of EMATs.

In common, EMATs are mainly composed of permanent magnets which provide bias magnetic fields and coils which generate eddy currents on the surface of conductive samples. To enhance the signal amplitudes, the most efficient method is to increase the eddy current density and the bias magnetic flux density. Silicon steel laminations were used as the back-plate, which increased the eddy current density and magnetic flux density by 25% and 3.9%, respectively. The results revealed that both the S0 and A0 mode Lamb waves were improved to certain extents [13]. A magnetic concentrator was added between the magnet and coil, which promoted the ability of EMATs for defect inspection [26,27].

Further studies focused on novel EMATs by exploiting various structures of coils and permanent magnets. The design of coils usually aims to increase the magnitude and optimize the distribution of eddy currents. Specially tuned double-coil EMATs were proposed to generate only symmetrical omnidirectional ultrasonic Lamb waves [28]. A contra-flexure coil and a cylindrical permanent magnet were employed to construct an omnidirectional EMAT for ultrasonic Lamb wave tomography imaging of defects in a metallic plate [29]. A novel design of SH EMAT, using a PPM array with a dual line-coil array instead of the common racetrack coil, was proposed to generate unidirectional SH waves by means of the longitudinally shifted and delayed line sources method [30].

As the effective distance between the north and south poles strongly affects the magnetic fields, a magnet array with adjacent magnets in opposite polarity provides larger magnetic fields than a conventional rectangular or cylindric magnet. A modified meander-line-coil (MLC) EMAT was presented, in which the two identical square magnets were laid alongside with opposite magnetization directions. The results showed that the signal-to-noise ratio was increased by a factor of 5.3 [25]. A novel EMAT consisting of nine magnets and two meander-line-coils was proposed for simultaneous detection of the horizontal and vertical components of Rayleigh waves [10]. A new magnet configuration, consisting of a cylindrical magnet surrounded by an annular one, was combined with spiral coils to achieve pure modes and improved amplitudes of ultrasonic waves [31,32]. An EMAT employing 12 axially polarized sector magnets that are arranged in a circle and a spiral coil was designed to generate omnidirectional SH0 waves [33].

Previous literature typically investigated the mode purity and amplitude enhancement only for either symmetric or antisymmetric mode Lamb waves. However, symmetric and antisymmetric modes are sensitive to different kinds of cracks [8,9]. There are few pieces of research on EMATs for selective generation between symmetric and antisymmetric ultrasonic Lamb waves. In this paper, we designed a type of Lamb wave EMAT that can not only enhance mode purity and amplitude but also selectively generate pure symmetric or antisymmetric mode waves at specific frequencies.

In this paper, the structure and working principles of the proposed EMAT are introduced. To further explain the mechanism of S0 and A0 mode Lamb waves, the proposed EMAT is divided into two simplified models. One mainly generates horizontal Lorentz forces and the other vertical Lorentz forces. Then two-dimensional FE simulation of the two simplified models is implemented to verify the mechanism of A0 and S0 mode waves. EMATs are fabricated and tested in the experiments to corroborate the capability of selectively generating pure mode waves with improved amplitude.

## 2. Design of the EMAT

**Figure 1:**
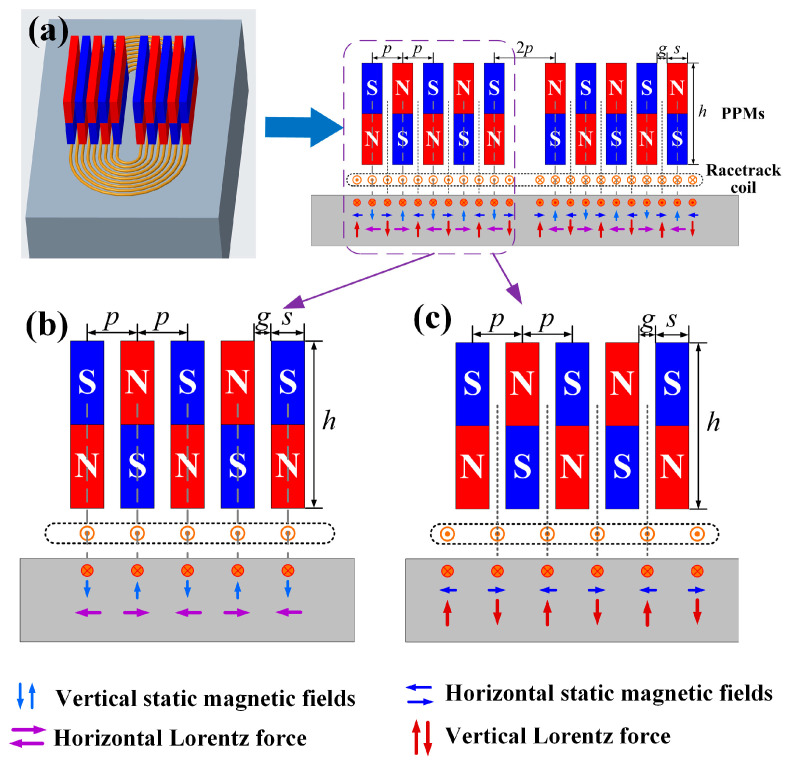
Schematics of the Lamb wave EMAT. (**a**) periodic permanent magnet EMAT. (**b**) WBM EMAT model, in which wires are aligned beneath the magnet units. (**c**) WBG EMAT model, in which wires are aligned beneath the gaps between magnets. *p* is the magnet spacing, *s* and *h* are, respectively, the width and height of the magnet units, and *g* is the gap between adjacent magnet units.

In general, Lorentz force, magnetostrictive force, and magnetizing force are the three coupling mechanisms for ultrasound generation and reception. For non-ferromagnetic samples, only the Lorentz force is taken into account [34]. Figure 1 shows the structure of the proposed EMAT for ultrasonic Lamb waves based on the Lorentz force mechanism. The alternating current ***J***_s_ in the racetrack coil generates a dynamic magnetic field ***B***_d_ and induces eddy currents ***J***_e_ in the skin layer of the conductive sample. The dynamic magnetic field and the eddy current are given by

(1) $$B_{d}=\nabla \times A,$$

and

(2) $$J_{e}=-\sigma \frac{\partial A}{\partial t},$$

respectively, where *σ* is the electrical conductivity of the sample and ***A*** is the magnetic vector potential. The periodic permanent magnets provide a static bias magnetic field ***B***_s_, which is expressed by

(3) $$B_{s}=\mu H+B_{r},$$

where *μ* is the permeability, ***H*** is the magnetic field intensity, and ***B***_r_ is the remanent magnetic flux density. Then Lorentz force ***F***_L_ is induced by the interaction between the magnetic field and eddy current, which is expressed as

(4) $$F_{L}=J_{e}\times (B_{s}+B_{d}).$$

Here, the dynamic magnetic field ***B***_d_ is, in general, negligibly small and can be omitted.

The structure of the developed PPM EMAT is different from the conventional SH wave EMAT, although both consist of a racetrack coil and a group of periodic permanent magnets. The magnet units in SH wave EMAT are arranged overhead the wires in the same direction as the excitation current. As a result, the Lorentz forces are tangential and vary with the polarities of magnet units. In Figure 1a, the magnet units are laid in the same direction as the wires. Then the direction of Lorentz forces is determined by the location of the wires. The wires beneath the magnet units mainly generate tangential Lorentz forces, and the wires beneath the gaps mainly generate normal ones, as shown in Figure 1a. It is inferred that the wires beneath the magnet units generate in-plane (IP) displacements and the wires beneath the gaps cause out-of-plane (OP) displacements. In order to distinguish the contribution of Lorentz force direction to the Lamb wave modes, the proposed EMAT is divided into two idealized categories. One is the wire-beneath-magnet (WBM) EMAT, as shown in Figure 1b, in which the wires are aligned with the magnet units. The other is the wire-beneath-gap (WBG) EMAT, as shown in Figure 1c, in which the wires are aligned with the gaps between magnet units.

In WBM EMAT, the wires and induced eddy currents are laid beneath the magnet units, and the efficient magnetic fields are perpendicular to the sample surface. This results in tangential Lorentz forces whose directions alternately change with the polarities of the magnet units. This type of EMAT can achieve purer S0 mode waves as compared with the conventional MLC EMAT, as already reported in [35]. In WBG EMAT, the wires and eddy currents are laid beneath the gaps between the magnets, and the efficient magnetic fields are parallel to the sample surface. The generated Lorentz forces are vertical, and the directions also alternately vary with the same period of the magnet array. It is inferred that this type of EMAT is suitable for generating antisymmetric mode Lamb waves that have dominant OP displacements. The WBM EMAT in Figure 1b, the WBG EMAT in Figure 1c, and the PPM EMAT in Figure 1a will be respectively researched in the following. The WBM and WBG EMATs are compared to verify the contribution of Lorentz forces with different directions to the Lamb wave modes. The PPM EMAT is researched to verify the performance improvement of the proposed design.

Furthermore, in the PPM EMATs shown in Figure 1, both the directions of tangential and normal Lorentz forces alternately change with the same period of PPMs, *p*. When the magnet spacing *p* is half of the wavelength *λ* of the targeted mode waves, the constructive interference gives rise to amplitude enhancement of Lamb waves by simply summing up the vibration caused by Lorentz forces [36,37,38]. In order to maximize the energy of the targeted mode waves, the center frequency of the excitation signal should be

(5) $$f_{c}=\frac{c_{p}}{\lambda }=\frac{c_{p}}{2p}=\frac{c_{p}}{2(s+g)},$$

where *f*_c_ and *λ* are the center frequency and wavelength of the targeted mode waves, *s* and *g* are the width and gap of magnet units, and *c*_p_ is the phase velocity of the targeted mode waves.

**Figure 2:**
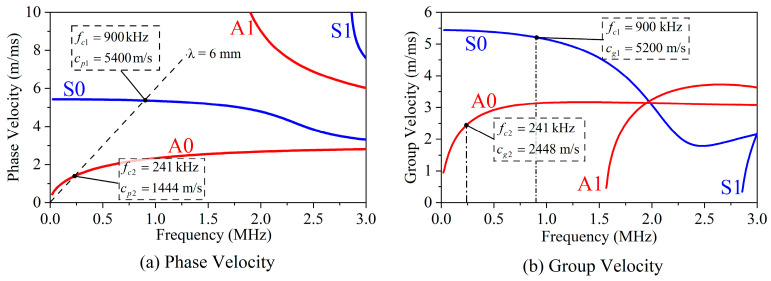
Lamb waves dispersion curves in a 1-mm-thick aluminium plate.

In this paper, a 1-mm-thick aluminium plate is employed as the sample. Magnet units (neodymium-iron-boron, Nd-Fe-B) have a size of 2 mm × 10 mm × 50 mm and a spacing *p* of 3 mm. Therefore, the wavelength of the targeted mode wave is 6 mm. Figure 2 shows the dispersion curves of ultrasonic Lamb waves in a 1-mm-thick aluminium plate. According to Equation (3), the theoretical center frequencies of S0 and A0 modes can be calculated to be *f*_c1_ = 900 kHz and *f*_c2_ = 241 kHz, respectively. The phase and group velocities of the S0 mode are *c*_p1_ = 5400 m/s and *c*_g1_ = 5200 m/s. The phase and group velocities of the A0 mode are *c*_p2_ = 1444 m/s and *c*_g2_ = 2448 m/s. This means that the output displacements of the WBM and WBG EMAT maximize at 900 kHz and 240 kHz, respectively. Then, the generated Lamb wave of the proposed EMATs in Figure 1a is investigated in the following simulations and experiments.

## 3. Simulation

**Figure 3:**
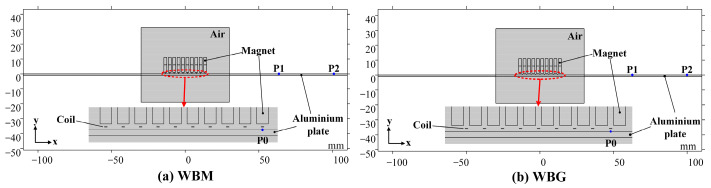
2-D FE simulation models of the WBM and WBG EMAT. (**a**) WBM model, **P0** (12, 0), **P1** (60, 0) and **P2** (100, 0). (**b**) WBG model, **P0** (13.5, 0), **P1** (60, 0) and **P2** (100, 0). **P0** is located on the sample surface and beneath the rightmost wires.

**Figure 4:**
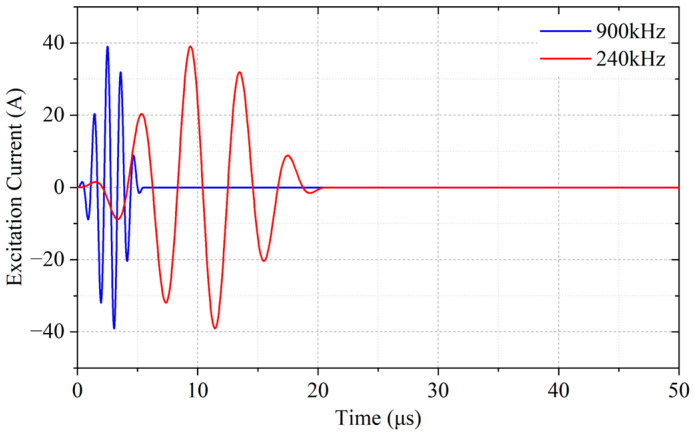
Excitation signals used in the simulations. The signal at 900 kHz is used in the WBM model, and the signal at 240 kHz is used in the WBG model.

In this section, two-dimensional finite element (FE) simulation models of the WBM and WBG EMATs were established using the COMSOL Multiphysics 5.5 software. Such parameters as the static magnetic fields, eddy currents, Lorentz forces, and displacements were comparatively studied with respect to their contribution to the generation of the symmetric and antisymmetric mode of ultrasonic Lamb waves. Each model consisted of a magnet domain, coil domain, sample domain, and air domain, as shown in [35]. As mentioned above, the widely used Nd-Fe-B magnets and aluminium plates are chosen in the simulations. The geometric and physical parameters are shown in Table 1. It is important to emphasize that the two models in Figure 3 have the same configuration of magnet units and aluminium plate and that the only difference is the position of wires. **P0** is a point on the top surface of the aluminium plate and beneath the rightmost wire. The coordinates of **P0** in the WBM and WBG models are (12, 0) and (13.5, 0), respectively. **P1** (60, 0) and **P2** (100, 0) are points on the top surface of the aluminium plate, which are used to observe the vibration and calculate the velocity of the ultrasonic wave. The pulse current signal in the excitation coil is a sinusoidal Hanning window signal with five cycles, as expressed by

(6) $$I=A_{0}\cdot \sin2\pi ft\cdot \left(1-\cos2n\pi ft\right),\;(t\leq nT),$$

where *A*_0_ is the amplitude of the excitation current, *f* the excitation frequency, *T* the period, and *n* the number of periods. The current signals at 900 kHz and 240 kHz are respectively used in the WBM and WBG models, as shown in Figure 4.

### 3.1. Static Magnetic Field

**Figure 5:**
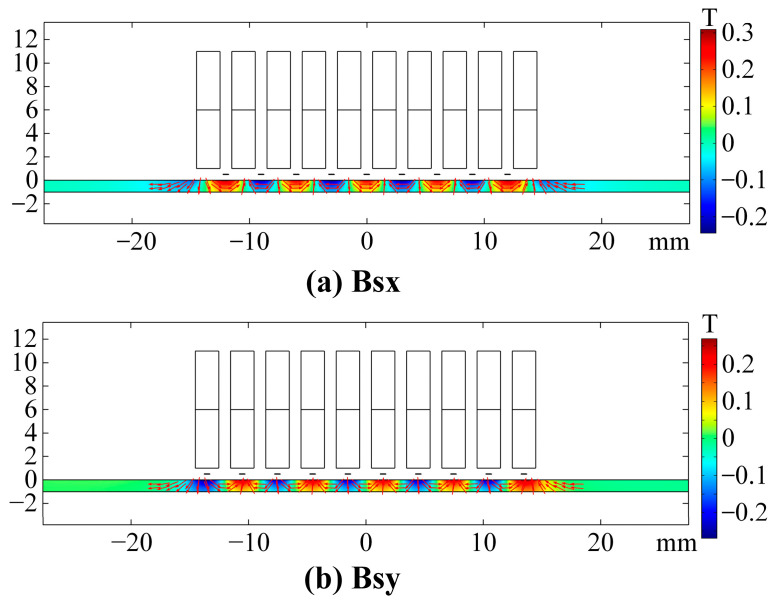
Distribution of the static magnetic flux density of periodic permanent magnets. (**a**) Vertical components. (**b**) Horizontal components.

**Figure 6:**
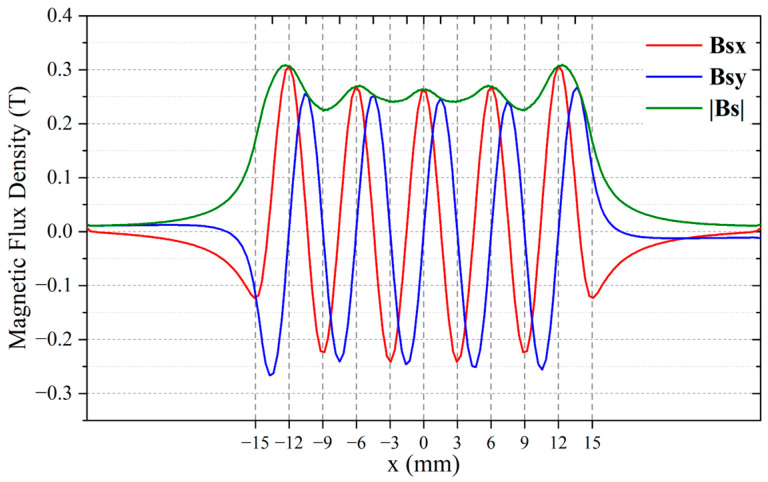
Distribution of the static magnetic flux density at the top surface of the aluminium plate.

The WBM and WBG models have identical periodic permanent magnet arrays. The only difference between the two models is the position of wires, which directly affects the magnitudes and directions of Lorentz forces. Figure 5 shows the static magnetic flux density **Bs** distribution in the aluminium plate. Figure 6 shows the variation of **Bs** components along the *x*-axis at the top surface of the aluminium plate. It is obvious that the vertical component *Bs*y reaches the maximum, and the horizontal component *Bs*x is close to zero at the center of the magnet units. At the gaps between magnets, *Bs*x reaches the maximum, and *Bs*y is close to zero.

### 3.2. Eddy Current

**Figure 7:**
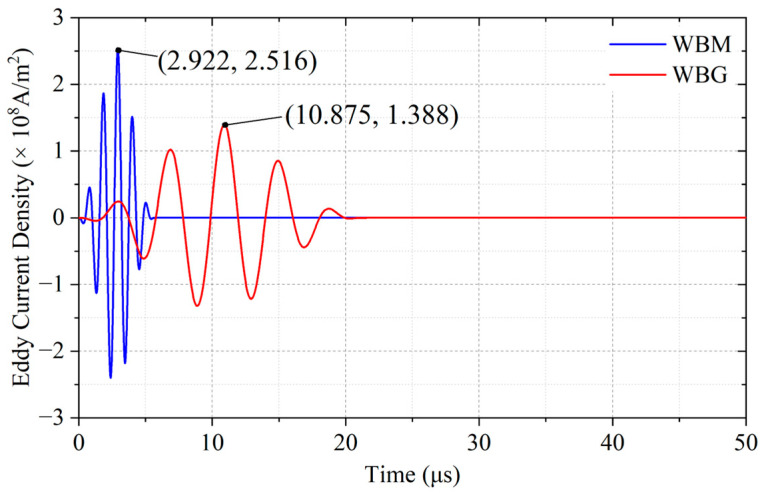
Eddy currents at **P0** for the WBM and WBG models.

**Figure 8:**
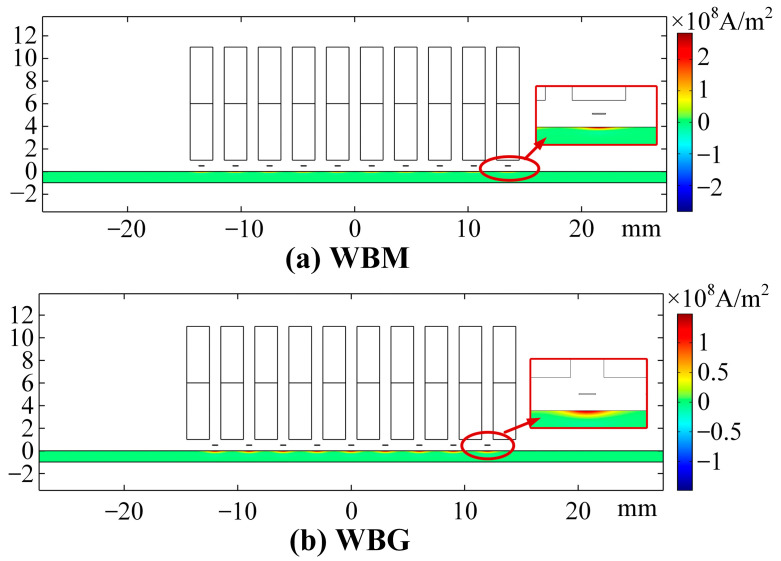
Eddy current density distribution of (**a**) WBM model at 2.922 μs and (**b**) WBG model at 10.875 μs.

The eddy current density *Ji*z at P0 is shown in Figure 7. In the WBM model, the eddy current reaches the maximum of 2.516 × 10^8^ A/m^2^ at 2.922 μs. Figure 8a shows the eddy current distribution in the WBM model at 2.922 μs. In the WBG model, the eddy current reaches the maximum of 1.388 × 10^8^ A/m^2^ at 10.875 μs. Figure 8b shows the eddy current distribution in the WBG model at 2.922 μs. Due to the different excitation frequencies, the eddy current in the WBM model has a larger maximum value and a smaller skin depth than those in the WBG model, as shown in Figure 8. The skin depth is expressed by

(7) $$\delta =\frac{1}{\sqrt{\pi f\mu \sigma }},$$

where *f* is the frequency of the excitation signal, *μ* and *σ* are the permeability and conductivity of the sample, respectively. The resulting *δ* of the WBM and WBG model is 0.0875 mm and 0.169 mm, respectively. The theoretical skin depth at 900 kHz is nearly half that at 240 kHz, which is in accordance with the simulation results.

### 3.3. Lorentz Force

**Figure 9:**
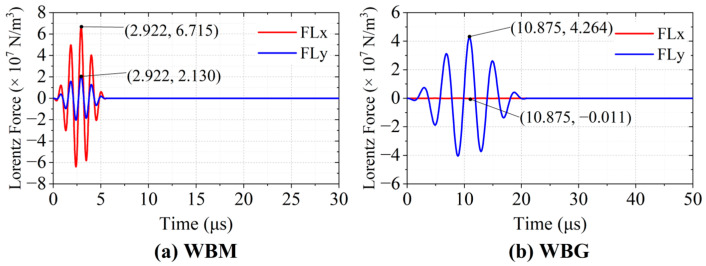
Lorentz forces at **P0** of the WBM and WBG models.

The Lorentz force density **FL** at **P0** is shown in Figure 9. It is obvious that the magnitudes of Lorentz forces at **P0** vary with the eddy currents. Different positions of the coils result in different directions of Lorentz forces. In the WBM model, the wires are aligned with the magnet units. It is the horizontal Lorentz forces *FL*x, resulting from the interaction between the vertical magnetic field and the eddy currents, that generate the Lamb waves. As shown in Figure 9a, the maximum values of *FL*x and *FL*y are 6.715 × 10^7^ N/m^3^ and 2.130 × 10^7^ N/m^3^, respectively. The ratio of the maximum values of *FL*x to that of *FL*y (η_WBM(x_y)_) is about 3.15.

The wires in the WBG model are aligned with the gaps between adjacent magnets. The Lorentz forces originated from the horizontal magnetic fields, and the eddy currents have vertical components larger than the horizontal ones. In Figure 9b, the maximum values of *FL*x and *FL*y are −0.011 × 10^7^ N/m^3^ and 4.264 × 10^7^ N/m^3^, respectively. The ratio of the maximum values of *FL*y to that of *FL*x (η_WBM(y_x)_) is about 388.

**Figure 10:**
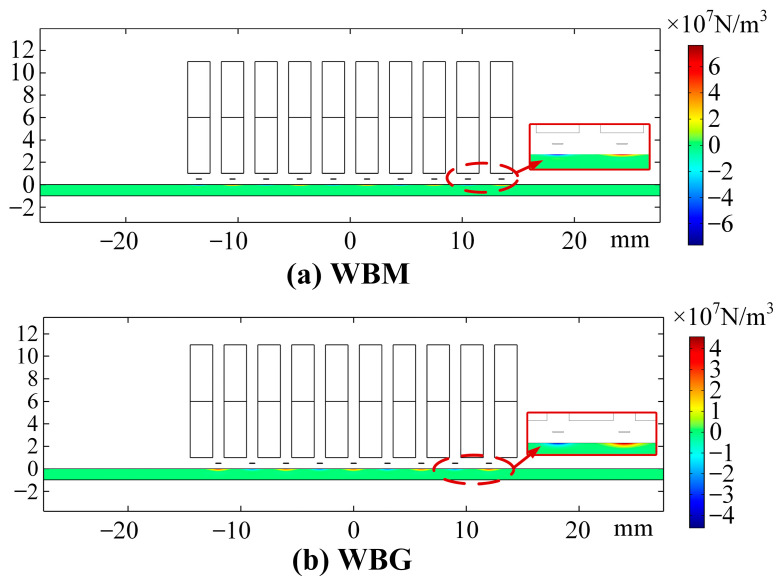
Lorentz force distribution. (**a**) Horizontal Lorentz force in the WBM model at 2.922 μs and (**b**) Vertical Lorentz force in the WBG model at 10.875 μs.

Figure 10 shows the Lorentz force distribution in the aluminium plate of the WBM model at 2.922 μs and the WBG model at 10.875 μs. Compared with those in the WBG model, the Lorentz forces in the WBM model are concentrated in a narrower area. This can be explained with the skin depth theory and the different excitation frequencies.

### 3.4. Lamb Wave Mode

**Figure 11:**
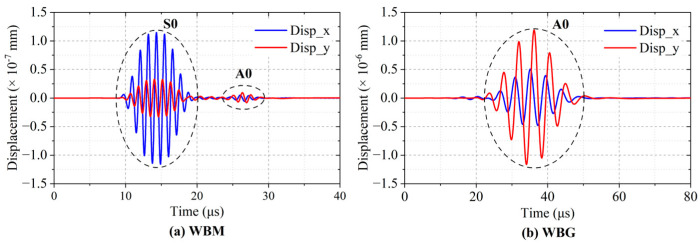
Horizontal and vertical displacements at **P1** (60, 0) of the WBM and WBG models.

**Figure 12:**
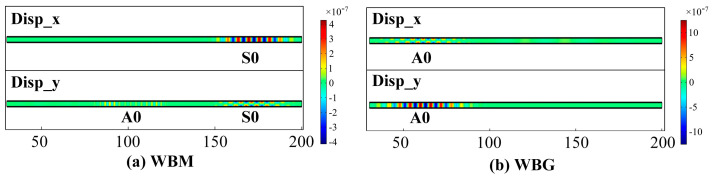
Displacement distribution of the WBM and WBG models at 36 μs.

**Figure 13:**
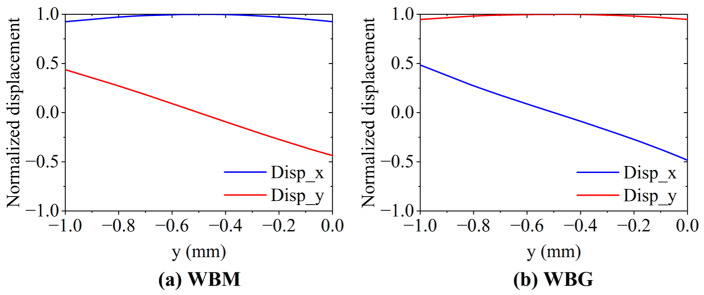
Normalized displacement on the line at x = 60 mm along the plate thickness in (**a**) the WBM model at 14.5 μs and (**b**) the WBG model at 32.5 μs.

Figure 11 shows the displacement at **P1** in the WBM and WBG models. In Figure 11a, the IP displacements are larger than the OP displacements, while in Figure 11b, the OP displacements are larger than the IP ones. This can be attributed to the Lorentz force distribution. Figure 12 and Figure 13 show the displacement distribution of the WBM and WBG models, respectively. In the wave of the WBM model, the IP displacement is symmetric about the center of the plate thickness and reaches the maximum at the center of the plate thickness, while the OP displacement is antisymmetric about the center of the plate thickness and is zero at the center of the plate thickness. In the wave of the WBG model, the IP displacement is antisymmetric, and the OP displacement is symmetric.

**Figure 14:**
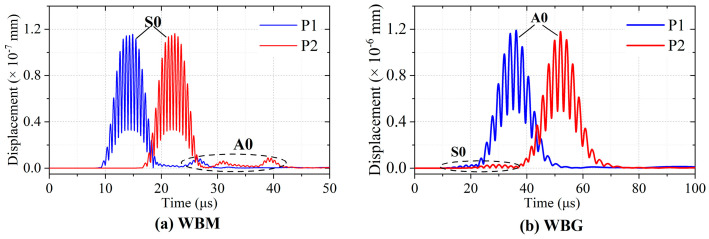
Total displacements at **P1** and **P2** of the WBM and WBG models.

Furthermore, the mode of the wave is verified by the wave velocity. Figure 14 shows the total displacements at **P1** and **P2**. The distance between **P1** and **P2** is 40 mm. In Figure 14a,b, the simulated velocity is 5333 m/s and 2526 m/s, close to the theoretical group velocity of S0 (5400 m/s) and A0 (2448 m/s) mode, respectively. This indicates that the WBM model mainly excites S0 mode Lamb waves, and the WBG model mainly excites A0 mode Lamb waves in the aluminium plate.

## 4. Experiment

**Figure 15:**
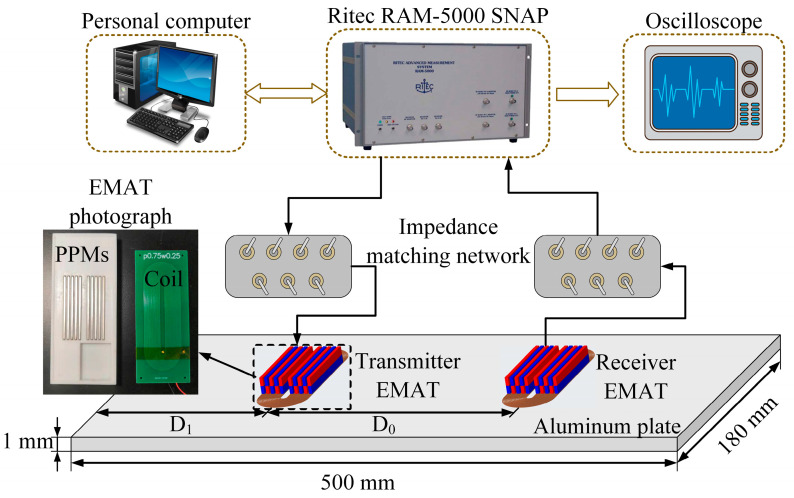
Schematic diagram of the experimental system.

In this section, EMATs were fabricated to implement the ultrasonic experiments. A schematic diagram of the experimental system is shown in Figure 15. The size of the aluminium plate is 500 mm × 180 mm × 1 mm. A pair of EMATs with identical structures is used as the transmitter and receiver. D_0_ is the distance between the transmitter and the receiver, and D_1_ is the distance from the transmitter to the left edge of the aluminium plate. The Ritec RAM-5000 SNAP system is employed to generate high-voltage pulse signals and to receive electrical signals controlled by means of a personal computer. Impedance-matching networks (TEM-128 Transmit EMAT Matching Network and REMP-128 Receive EMAT Matching Network) are added to the transmitter and receiver to improve the energy conversion efficiency of the transducers. A digital oscilloscope (Tektronix MDO3012 Mixed Domain Oscilloscope) is adopted to display and store electrical signals.

### 4.1. Verification of Wave Mode

**Figure 16:**
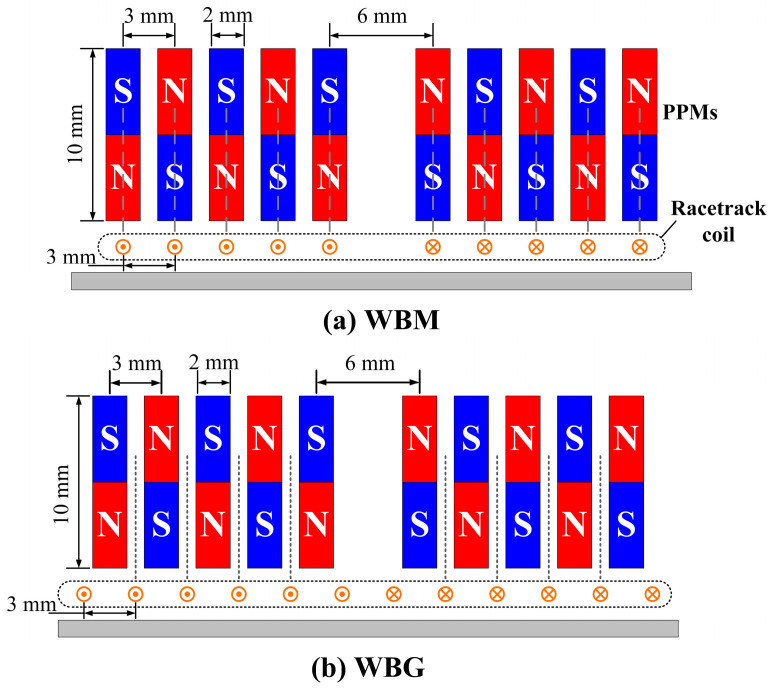
Structure diagrams of the WBM and WBG EMATs in experiments.

In this experiment, WBM and WBG EMATs with coils of different sizes were fabricated. The cross-sections are schematically shown in Figure 16. Different from the simplified simulation models, racetrack coils manufactured with printed circuit board (PCB) technology are used as exciting coils to ensure processing accuracy. Two groups of PPMs are adopted to fully utilize the wires of the racetrack coils. It is noted that the distance between the middle two magnet units is set equal to the wavelength to avoid destructive interference of Lorentz force distribution. The size of Nd-Fe-B magnet units is 2 mm × 10 mm × 50 mm. Racetrack coils with different sizes were manufactured, aiming to investigate the effect of the width of wires on the EMAT performance. The dimensional parameters of the coils are shown in Table 2.

**Figure 17:**
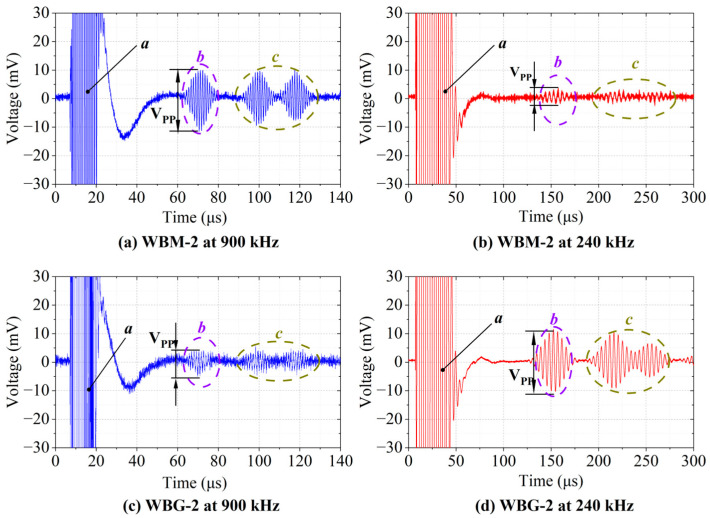
Signals received by the WBM-2 and WBG-2 EMATs. (**a**) Signal of WBM−2 EAMT at 900 kHz. (**b**) Signal of WBM-2 EMAT at 240 kHz. (**c**) Signal of WBG-2 EMAT at 900 kHz. (**d**) Signal of WBG-2 EMAT at 240 kHz. Packets *a* are the crosstalk signals, packets *b* are the direct waves, and packets *c* are echoes reflected by the left and right edges of the aluminium plate. V_PP_ is the peak-to-peak value of packets *b*.

Five-cycle tone burst signals modulated by a Hanning window at 900 kHz and 240 kHz were used as the excitation signals. The received signals of WBM-2 and WBG-2 EMATs are shown in Figure 17 for D_0_ of 300 mm and D_1_ of 75 mm. The modes of the received signals can also be determined by the calculated wave velocity. The experimental results of the wave velocities at 900 kHz and 240 kHz are 5024 m/s and 2366 m/s, respectively. Compared with the theoretical group velocity, it is proved that the WBM-2 and WBG-2 EMATs are prone to generate and receive S0 mode Lamb waves at 900 kHz and A0 mode Lamb waves at 240 kHz, respectively.

Comparing Figure 17a,b, one can see that the amplitudes of Lamb waves at 900 kHz are larger than those at 240 kHz. This indicates that WBM-2 EMAT is more suitable for S0 than for A0 mode ultrasonic Lamb waves. On the contrary, the amplitudes of Lamb waves in Figure 17d are larger than those in Figure 17c, which illustrates better performance on A0 mode waves of WBG-2 EMAT.

**Figure 18:**
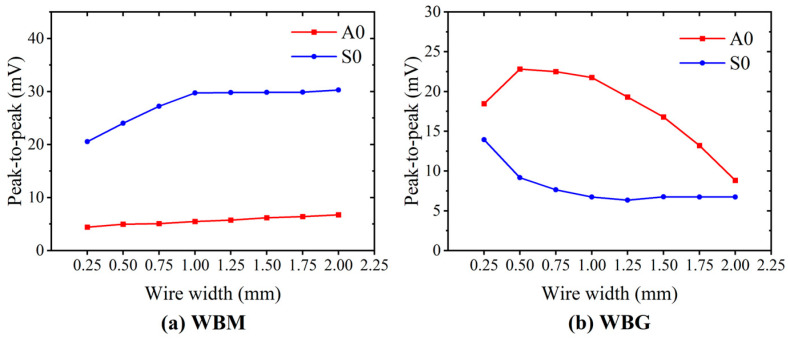
Effect of the wire width on the amplitude of the received signals.

The amplitude (V_PP_) of the direct waves received by the WBM and WBG EMATs with different wire widths is shown in Figure 18. In WBM EMATs, the amplitude of S0 modes is always larger than that of A0 modes. While in WBG EMATs, the amplitude of S0 modes is always smaller than that of A0 modes. Moreover, in Figure 18a, the amplitude of both S0 and A0 mode waves received by WBM EMATs increases with the increasing wire width. The increase in the amplitude of the S0 mode is more pronounced than that of the A0 mode for wire width below 1 mm. Then, the amplitude of the S0 mode remains almost constant, while that of the A0 mode still slightly increases with increasing wire width. The dependence of the Lamb wave amplitude on the wire width is quite different for the WBM EMAT (Figure 8b). The amplitude of S0 mode first decreases with increasing wire width and then reaches a stable value above a wire width of 1 mm. The amplitude of the A0 mode increases with increasing wire width when the latter is smaller than 0.5 mm and then starts to decrease above a wire width of 0.5 mm.

### 4.2. Performance of Proposed EMAT

**Figure 19:**
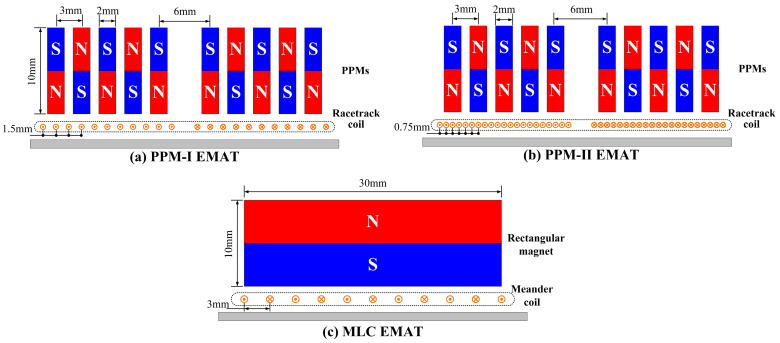
Structure diagram of developed PPM EMATs and conventional MLC EMAT. (**a**) PPM-Ⅰ EMAT, the coil is a racetrack coil with wire spacing of 1.5 mm. (**b**) PPM-Ⅱ EMAT, the coil is a racetrack coil with wire spacing of 0.75 mm. (**c**) MLC EMAT, the coil is a meander coil with wire spacing of 3 mm.

The EMATs, in which the wires are located beneath either the magnet units or gaps between adjacent magnets, have been discussed above. Then the EMATs with wires laid uniformly beneath both the magnets and gaps were fabricated. Conventional MLC EMATs, consisting of a rectangular magnet and a meander coil, were also fabricated for comparison. The structures are schematically shown in Figure 19. The size of the rectangular magnet is 30 mm × 10 mm × 50 mm. Table 3 shows the dimensional parameters of the racetrack and meander coils.

**Figure 20:**
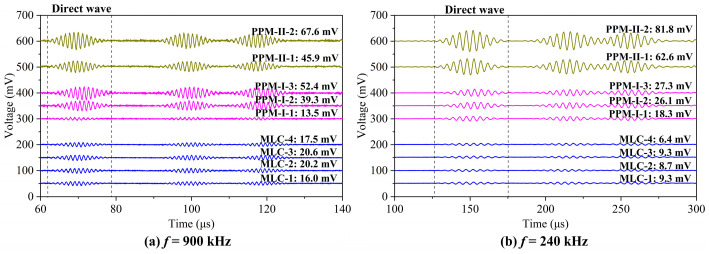
Received signals of the MLC and PPM EMATs at (**a**) 900 kHz and (**b**) 240 kHz.

**Figure 21:**
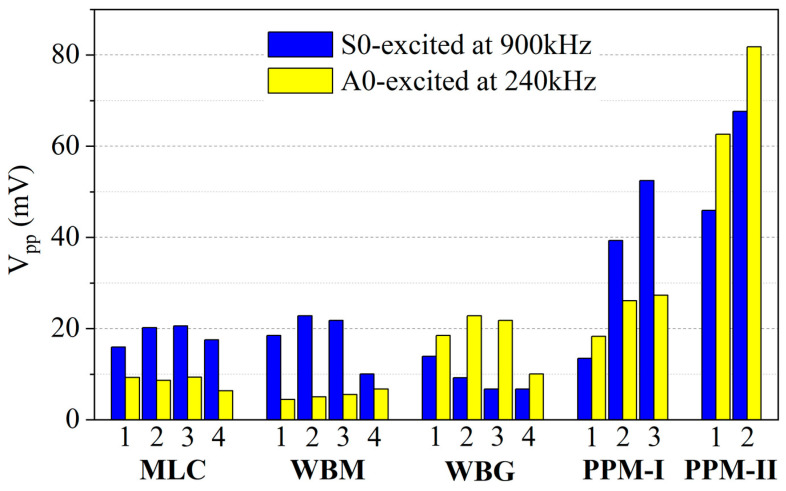
Comparison of the signal amplitudes received by means of different types of EMATs.

The signals received by the three types of EMATs at frequencies of 900 kHz and 240 kHz are shown in Figure 20. It can be seen that the coexistence of horizontal and vertical Lorentz force does not affect the purity of A0 and S0 mode Lamb waves due to constructive and destructive interference. S0 and A0 modes dominate the Lamb waves excited at 900 kHz and 240 kHz by each type of EMAT, respectively. The peak-to-peak values (V_pp_) of the direct wave packets in the received signals are calculated and shown in Figure 21.

Among the MLC transducers, MLC-3 has the best performance for the generated Lamb waves of the A0 and S0 modes. The amplitudes of the S0 and A0 modes in the MLC-3 signals are 20.6 mV and 9.3 mV, respectively. This is because the Lorentz forces are dominated by the horizontal ones in the center area of the rectangular magnet, which results in IP displacements significantly larger than the OP ones.

Comparing the signals received by the PPM-Ⅰ and -Ⅱ EMATs, one can see that the increase in the wire width and wire density leads to a significant enhancement of the amplitude. It can be attributed to the increase in eddy current density. PPM-Ⅱ-2 gives the highest amplitude both for the S0 (67.6 mV) and A0 (81.8 mV) modes. The ratio of the largest S0 mode amplitude of the proposed PPM EMAT to that of the conventional MLC EMAT (η_S0_) is about 328%, and the ratio of the largest A0 mode amplitude of the proposed PPM EMAT to that of the conventional MLC EMAT (η_A0_) is about 880%.

It is obvious that the developed periodic permanent magnet EMAT has a larger enhancement for the A0 mode than the S0 mode. This is because the conventional MLC EMAT mainly generates vertical magnetic fields at the central area of the rectangular magnet, and the horizontal magnetic fields only exist in the peripheral area due to the edge effects. It is the horizontal Lorentz force, resulting from the vertical magnetic fields, that generates the Lamb waves. In the developed PPM EMAT, the vertical and horizontal magnetic fields distribute mainly beneath the magnets and gaps, respectively. Both horizontal and vertical Lorentz forces contribute to the generation of Lamb waves. Moreover, the larger wire density gives rise to an increase in the eddy current density, which further enhances the Lorentz forces. This is the reason for the significant enhancement of the developed EMATs on generated Lamb waves.

## 5. Conclusions

With the aim of improving the energy conversion efficiency and the signal amplitude of Lamb wave EMATs for non-ferromagnetic materials, this article proposes a Lamb wave EMAT design for ultrasonic inspection of non-ferromagnetic plates, which can selectively generate pure symmetric or antisymmetric mode waves at specific frequencies. The periodic permanent magnet EMAT consists of a racetrack coil and a group of PPMs. The racetrack coils can increase the eddy current density by increasing the wire density, and the periodic permanent magnets provide magnetic fields with a spatially periodic distribution. The directions of the generated Lorentz forces alternately change with the polarities of the magnet units. When the magnet spacing is half the wavelength of the generated Lamb waves, the corresponding mode reaches the maximum amplitude due to constructive interference.

The proposed PPM EMAT was divided into two simplified models according to the directions of Lorentz forces, aiming to analyze the generation mechanism of symmetric and antisymmetric modes of Lamb waves. 2D FE simulations were established in order to compare the eddy currents, bias magnetic fields, Lorentz forces, and displacement of simplified WBM and WBG models. Several groups of transducers were manufactured and tested in the experiments. The results prove that the tangential and normal Lorentz forces mainly contribute to symmetric and antisymmetric modes of ultrasonic Lamb waves, respectively. When the wires of the racetrack coil are laid uniformly beneath the magnets and gaps of the PPMs, the proposed EMAT can generate Lamb waves with pure mode and enhanced amplitude at specific frequencies. The amplitude increases with the increasing width and density of the wires. Compared with the case of conventional MLC EMAT, the amplitudes of S0 and A0 mode waves can be increased to 328% and 880%, respectively. In the future, omni-directional ultrasonic Lamb wave EMAT will be developed from the proposed PPM EMAT to achieve two-dimensional imaging of defects in plate structures.

## Figures and Tables

**Table 1:** Geometric and physical parameters of simulation models.

Component	Parameter	Value
Air	Width	60 mm
	Height	50 mm
	Relative permeability	1
	Conductivity	0 S/m
	Relative dielectric constant	1
Magnet	Width of magnet units	2 mm
	Height of magnet units	10 mm
	Magnet unit spacing	3 mm
	Relative permeability	1.04
	Conductivity	6.25 × 10^5^ S/m
	Relative dielectric constant	1
	Residual magnetic flux density	1.3 T
Coil	Width of wires	0.5 mm
	Thickness of wires	0.035 mm
	Wire spacing	3 mm
	Distance between magnet and coil	0.5 mm
	Distance between coil and plate	0.5 mm
	Relative permeability	1
	Conductivity	5.998 × 10^7^ S/m
	Relative dielectric constant	1
Aluminium	Width	400 mm
	Thickness	1 mm
	Relative permeability	1
	Conductivity	3.774 × 10^7^ S/m
	Relativity dielectric constant	1
	Density	2700 kg/m^3^
	Young’s modulus	7.0 × 10^10^ Pa
	Poisson’s ratio	0.33

**Table 2:** Dimensional parameters of coils in WBM and WBG EMATs.

Model		Wire Spacing	Wire Width
WBM	1	3.00 mm	0.25 mm
	2		0.50 mm
	3		1.00 mm
	4		2.00 mm
WBG	1	3.00 mm	0.25 mm
	2		0.50 mm
	3		1.00 mm
	4		2.00 mm

**Table 3:** Dimensional parameters of coils in PPM and MLC EMATs.

Model		Wire Spacing	Wire Width
PPM-Ⅰ	1	1.50 mm	0.25 mm
	2		0.50 mm
	3		1.00 mm
PPM-Ⅱ	1	0.75 mm	0.25 mm
	2		0.50 mm
MLC	1	3.00 mm	0.25 mm
	2		0.50 mm
	3		1.00 mm
	4		2.00 mm

## Data Availability

Not applicable.
